# Determination of Calcium in Meat Products by Automatic Titration with 1,2-Diaminocyclohexane-N,N,N’,N’-tetraacetic Acid

**DOI:** 10.3390/molecules28186592

**Published:** 2023-09-13

**Authors:** Alexander Shyichuk, Maria Kowalska, Iryna Shyychuk, Jan Lamkiewicz, Dorota Ziółkowska

**Affiliations:** Faculty of Chemical Technology and Engineering, Bydgoszcz University of Science and Technology, Seminaryjna 3, 85-326 Bydgoszcz, Poland

**Keywords:** calcium, mechanically separated meat, mechanically deboned meat, skin, cartilage, tendon

## Abstract

Mechanically separated meat (MSM) is a by-product of the poultry industry that requires routine quality assessment. Calcium content is an indirect indicator of bone debris in MSM but is difficult to determine by EDTA titration due to the poor solubility of calcium phosphate. Therefore, 1,2-diaminocyclohexane-N,N,N’,N’-tetraacetic acid was used instead, which has two orders of magnitude higher affinity for calcium ions. In addition, the auxiliary complexing agents triethanolamine and Arsenazo III, an indicator that is sensitive to low calcium concentrations, were used. Automatic titration endpoint detection was performed using an immersion probe at 660 nm. It has been shown that the color change in Arsenazo III can also be read with an RGB camera. The CDTA titration procedure has been tested on commercial Bologna-type sausages and the results were in line with AAS and ICP reference data. The content of calcium in sausages turned out to be very diverse and weakly correlated with the content of MSM. The tested MSM samples had a wide range of calcium content: from 62 to 2833 ppm. Calcium-rich poultry by-products include fat and skin (115 to 412 ppm), articular cartilage (1069 to 1704 ppm), and tendons (532 to 34,539 ppm). The CDTA titration procedure is fully suitable for small meat processing plants due to its simplicity of use and low cost.

## 1. Introduction

Routine quantification of calcium is required for meat products containing mechanically separated meat (MSM). MSM is the product of mechanical de-boning in the poultry and pork industries [1,2]. Due to the extensive destruction of muscle tissue, MSM carries with it an increased bacterial load and accelerated fatty acid oxidation [1,3,4]. The degree of muscle tissue damage correlates with the pressure applied, which is why there are two types: low-pressure MSM and high-pressure MSM [5]. The direct method for assessing the damage to muscle and bone tissue is the histological investigation of meat slices stained with haematoxylin/eosin [6,7]. Bone particles are usually stained with Alizarin Red and cartilage particles with Alcian Blue [8,9]. The number of meat samples for histological examination should be large enough to exclude the possibility of false-negative results. Bone fragments in meat can also be detected by X-ray microtomography [7,10] and ESR spectroscopy [8]. The type of MSM can be identified by measuring the density and velocity of ultrasound waves [11,12]. 

Useful indicators of MSM quality are chemical components such as calcium [13,14,15], ash [15], iron [13,16], and collagen [17,18]. Calcium content is considered the most appropriate of these chemical indicators for distinguishing between low-pressure and high-pressure MSM [5]. Food authorities, the EFSA and the USDA, have set the threshold levels of calcium at 1000 ppm and 1300 ppm, respectively [5,19].

Calcium content in MSM and meat products can be determined by various methods, each of which has its advantages and disadvantages. Titration with ethylenediaminetetraacetic acid (EDTA) is inexpensive but has indistinct endpoints due to the poor solubility of calcium phosphate from bone material. To overcome this problem, a back-titration procedure is used [20,21]. Atomic absorption spectroscopy (AAS) provides a reliable determination of calcium [13] but uses large amounts of gasses. In addition, an increased concentration of phosphate ions can interfere with the results as undissociated calcium phosphates are formed in the AAS flame, thus reducing the analytical signal. Phosphate interference can be suppressed by adding lanthanum ions to the solution under analysis. Inductively coupled plasma optical emission spectroscopy (ICP-OES) and inductively coupled plasma mass spectrometry (ICP-MS) have no interference from phosphate ions because they operate at much higher temperatures [22]. These techniques enable the simultaneous determination of multiple analytes and are often used to provide reference data [23,24,25]. Ion chromatography also allows the simultaneous determination of calcium and magnesium, which is useful for the reliable detection of MSM [14]. 

Since all of the above-mentioned techniques are for aqueous solutions, the samples must be prepared by acid digestion. Spectral techniques therefore tend to be more advantageous as they require little or no preparation. Laser-induced breakdown spectroscopy (LIBS) uses very high temperatures at the ablation spots so phosphates do not interfere [23,24]. The advantage of LIBS is that it can be used for quick at-line measurements without sample preparation; however, heterogeneous meat samples cause a great deal of noise in the LIBS spectra. The normalization of calcium spectral lines against potassium lines along with partial least squares modeling allows three levels of calcium content to be distinguished [23]. 

Raman spectroscopy has the advantage of not requiring any sample preparation and of recording spectra from a short distance. Raman spectra of fresh poultry meat, together with multiplicative signal correction and principal component analysis (PCA), enable the quantification of calcium with a coefficient of determination R^2^ of 0.775 [15]. However, detailed insight into principal component loadings suggests that the phosphate band is the most important signal correlated with calcium and ash percentages. This is fully understandable because MSM contains calcium mainly in the form of hydroxyapatite. Therefore, Raman spectroscopy is not suitable for the determination of calcium in commercial meat products, which often contain phosphate additives. Total reflection X-ray fluorescence (TXRF) requires little preparation of meat samples, though heterogeneous samples introduce additional inaccuracy due to the small spot of the X-ray beam [16]. Table 1 summarizes the key features of the above-mentioned methods. Due to the large variety of meat matrices, it is generally recommended to measure calcium by several different methods [5]. 

The literature data clearly indicate that calcium content alone does not reliably distinguish low-pressure MSM from minced meat products [5,14]. Detection reliability can be improved by using two or more variables: Ca and Mg [14]; Ca, Fe, and K [16]; or Ca, ^90^Sr, ^88^Sr, and ash [6]. The only PCA approach with a large set of 15 variables (Ca, Mo, Co, Ba, Sr, Ni, Se, Sn, Ho, Lu, As, Li, Pb, Tl, and Fe) provides fully reliable MSM detection in meat products [25]. Nevertheless, calcium content remains one of the best indicators of the quality of meat products. This is because meat products often contain other calcium-rich additives, such as chicken fat (150–400 ppm), whey protein (~470 ppm), soy protein (~1780 ppm), articular cartilage (~3800 ppm), milk powder (~9100 ppm), etc. Thus, an increased calcium content in a meat product indicates a higher amount of non-meat additives. 

**Figure 1:**
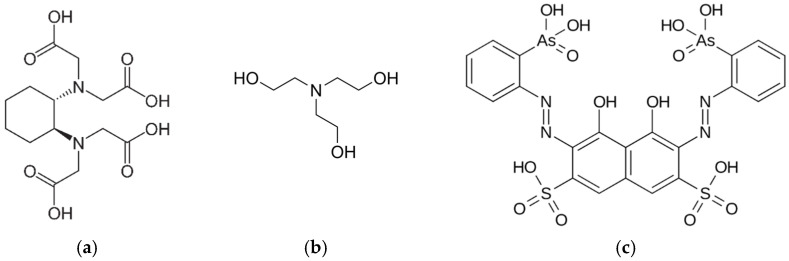
Structural formulae of (**a**) CDTA, (**b**) TEA, and (**c**) Arsenazo III.

Complexometric titration is a simple and reliable technique for calcium determination. However, for MSM analysis, the titration endpoint can be indistinct due to the poorly soluble calcium phosphates. At the step of sample preparation, the calcium hydroxyapatite contained in the bone particles is dissolved by acid digestion. In the next titration step, calcium phosphates are re-formed due to the alkaline pH needed for the complexation reaction. Suspended calcium phosphate particles react slowly with EDTA and therefore the color change is gradual. To improve endpoint detection, we propose using trans-1,2-diaminocyclohexane-N,N,N’,N’-tetraacetic acid (AKA cyclohexane trans-1,2-diamine tetra-acetate, CDTA) as the complexing reagent. Figure 1a shows the structural formula of CDTA. CDTA binds calcium ions more strongly than EDTA: the corresponding pK_a_ values are 15.0 and 12.4, respectively [26]. Due to its high complexing properties, CDTA is widely used in plant research to bind calcium ions that cross-link pectin polysaccharides in cell walls. A typical pectin fractionation procedure involves sequential extraction with water, a buffered CDTA solution, and an alkaline solution, yielding water-soluble pectin, chelate-soluble pectin, and alkali-soluble pectin, respectively [27,28,29,30,31,32,33,34]. CDTA is also used for the sequestration of polyvalent cations in fluoride ion determination with ion-selective electrodes [26,35].

CDTA was once studied as a reagent for determining calcium and magnesium in the presence of phosphate ions [36]. It was noted that direct titration of CDTA should be slow, especially near the endpoint, to ensure equilibrium with calcium phosphate, which is insoluble at pH > 12. Therefore, back titration is used to minimize tedious manual work [36]. On the other hand, two-step titration is known to be less accurate. Fortunately, back-titration is not currently needed as the dosing of the solution is performed by an automatic titrator. Therefore, this article is about automatic direct titration of CDTA. Another approach to reduce calcium phosphate precipitation and thus improve endpoint detection is to use triethanolamine (Figure 1b) as an auxiliary complexing agent [37]. Triethanolamine (TEA) forms mild complexes with calcium ions and modifies the surface of poorly soluble calcium salts. This effect is well known in the cement industry, which uses triethanolamine to improve clinker grinding and mortar hydration [38,39,40]. This work confirmed the positive effect of triethanolamine on endpoint detection. 

Phosphate ions compete with color indicators for calcium ions. Phosphate was found to interfere with the murexide indicator, but not with calcein [36]. However, because calcein is a fluorescent indicator that changes its emission but not its absorption of light, it cannot be used with the optical immersion probe needed for automatic titration. Thus, the indicator used in this work was Arsenazo III (2,2′-(1,8-dihydroxy-3,6-disulfo-2,7-naphthalene-bis[azo])dibenzenearsonic acid) with the structure shown in Figure 1c. Arsenazo III has a fairly high affinity for calcium and is used to quantify micrograms of calcium in biological samples [41,42,43]. 

The overall result of these improvements is a robust complexometric method well suited for the determination of calcium in meat products. The advantages of the complexometric method (small amounts of reagents, no derivatization, and no toxic reagents) fit well into the principles of Green Analytical Chemistry. In addition, automatic titrators are simple to use and do not require highly trained personnel. All these features make the complexometric titration method perfect for small meat processing plants. 

## 2. Results and Discussion

### 2.1. Optimization of the CDTA Titration Method 

#### 2.1.1. Selection of Optimal Wavelength and Indicator Concentration

**Figure 2:**
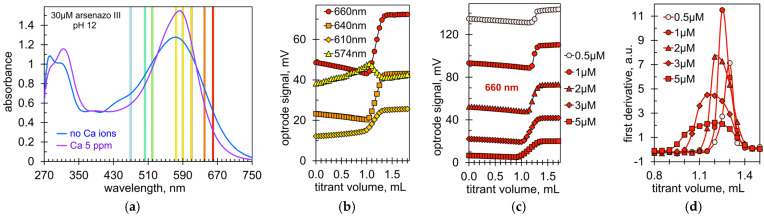
(**a**) Spectral changes of Arsenazo III in the presence of calcium ions. The operating wavelengths of the Optrode LEDs are indicated by the correspondingly colored bands. (**b**) Optrode signals at different wavelengths during CDTA titration of Ca model solutions. Arsenazo concentration is 2 μM. (**c**) Optrode signal at 660 nm vs. CDTA volume at different concentrations of Arsenazo III. (**d**) First derivatives of graphs (**c**).

Figure 2a shows the UV–vis spectra of Arsenazo III solution in the absence and presence of calcium ions. Spectral changes occur in the ranges of 270–360, 420–520, 550–610, and 630–740 nm. However, changes in absorbance are rather moderate (10% to 30% of initial values) and there are no distinct wavelengths that are best for direct measurements. That is why spectrophotometric determination of calcium with Arsenazo III uses a ratiometric approach at two wavelengths: 660 and 700 nm [42,43]. Thus, the selection of the optimal working wavelength for an immersion optical probe is no trivial task. Most Optrode operating wavelengths correspond at first glance to the spectral changes of Arsenazo III (Figure 2a). Experiments have shown that the signal changes at the wavelengths of 470, 502, 520, and 590 nm are too small. Suitable wavelengths turned out to be 574, 610, 640, and 660 nm. The most suitable wavelength is 660 nm, which provides the largest signal jump at the titration endpoint (Figure 2b).

Figure 2c shows titration graphs at different concentrations of the indicator. The Optrode signal at 660 nm depends on the absorption of red light by the indicator solution. Thus, as the concentration of Arsenazo III increases, the absorption of red light increases and the initial voltage signal decreases. The magnitude of the signal jump at the endpoint also depends on the indicator concentration but in a non-monotonic manner. The largest signal jump occurs at concentrations of 2 and 3 μM (Figure 2b,c). The first derivative plots show that the sharpest change occurs at the indicator concentration of 1 μM (Figure 2d). The conclusion is that the optimal concentration of Arsenazo III is in the range of 1 to 3 μM. However, a concentration of 3 μM is more favorable for the automatic titrator, as the higher voltage jump it provides is beneficial for the titrator setup. 

#### 2.1.2. Reduction in Interference Caused by Phosphate Ions 

**Figure 3:**
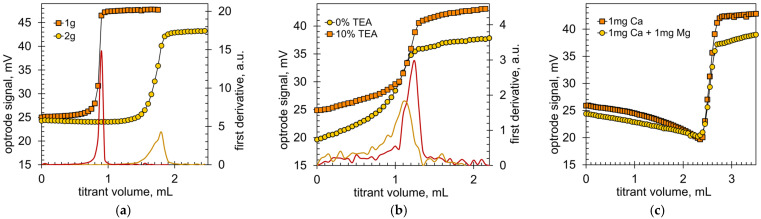
(**a**) Titration graphs obtained during the analysis of 1 g and 2 g samples of brand A chicken sausage. (**b**) The influence of TEA on the shape of the titration graph when analyzing 1 g of brand I turkey sausage. Smooth lines are the first derivative graphs of titration curves. (**c**) Titration graphs obtained with model solutions containing Ca and Mg ions and 10% TEA at a pH of 12.

CDTA and Arsenazo III are strong complexing agents for calcium ions but cannot completely overcome phosphate competition. Figure 3a shows the titration graphs obtained when analyzing a commercial chicken sausage. According to the label, brand A sausage contains soy protein and 61% MSM of chicken meat and skin. The calcium content was determined to be 339 ppm, which is higher than the typical 50–80 ppm in pure meat [44,45,46]. This high calcium content suggests the presence of bone material and therefore phosphate ions. Figure 3a shows that the sample size affects the results of the analysis. As the mass of the sample increases, the final Optrode voltage decreases, which indicates an increase in the turbidity of the solution. This is due to the formation of more calcium phosphate. The reaction of CDTA with suspended calcium phosphate particles is slow; therefore, the titration graph is less sharp and the derivative peak is wider (Figure 3a). The conclusion is that the optimal sample mass depends on the type of meat product under analysis. With increased bone content, a lower sample mass can provide more accurate endpoint detection.

Figure 3b shows the titration graphs obtained when analyzing brand I turkey sausage. The sausage label declares a content of 26.3% turkey MSM and 45.4% turkey meat. The amount of MSM is lower than in brand A sausage while the calcium content was measured as 472 ppm, which is higher than in brand A sausage. The brand I MSM probably contained more bone material. The justification for this thesis is the shape of the titration graph, which is much worse than for brand A (Figure 3a). The obvious reason may be a higher content of bone debris, but an additional reason may be phosphate additives. Food-grade phosphate salts are widely used in meat products to improve water holding [47]. Typical MSM has a reduced water-holding capacity due to the presence of bone and cartilage material and so a higher amount of phosphate salts is required [48,49]. It was found that in such a difficult case, the addition of triethanolamine improves the shape of the titration graphs (Figure 3b). 

TEA is known, as a mild complexing agent, to increase the dispersibility of hydroxyapatite when forming coatings on metal substrates [50,51]. Figure 3b clearly shows that the Optrode voltage increases in the presence of TEA. This is due to the reduced turbidity of the solution since a finer suspension is formed. The first derivative plot clearly shows that color change occurs over a narrower range of the titrant volume (Figure 3b) and thus, the addition of TEA results in a more pronounced endpoint. It is worth noting that the addition of TEA does not worsen the sequestration of magnesium ions at a strongly alkaline pH of 12. Figure 3c confirms that the titration endpoint does not change in the presence of magnesium ions.

#### 2.1.3. Validation of the CDTA Titration Method

For model solutions containing 0.5 mg of calcium ions, the RSD value was found to be 1.6%. For commercial meat products, the RSD values turned out to be higher (Table 2). Sources of additional inaccuracy are the mineralization and aliquot sampling steps. In addition, some solutions of meat products are turbid, making the endpoints less clear. The LOD values were 0.024 mg, 0.057 mg, and 0.078 mg for the model solution, brand B chicken sausage, and chicken meat, respectively. The corresponding LOQ values were 0.08 mg, 0.19 mg, and 0.26 mg calcium, respectively. The upper limit of the linearity range is not strictly defined as it depends on the turbidity of the solution. In order not to prolong the titration procedure, it is better to select a sample mass for which the amount of calcium does not exceed 3 mg. The 94% recovery value was determined by spiking the brand B chicken sausage sample with 1 mg of calcium. 

**Figure 4:**
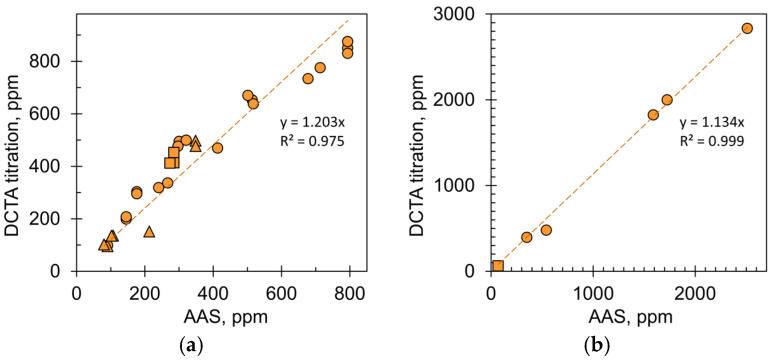
Correlation between complexometric titration and AAS measurements for (**a**) Bologna-type sausages and (**b**) MSM samples. The samples are (●) chicken, (■) turkey, and (▲) pork meat products.

Complexometric titration results were compared with AAS reference values (Figure 4). The coefficient of determination had quite high values: 0.975 (Figure 4a) and 0.999 (Figure 4b). This proves that the CDTA titration results are consistent with the AAS measurements. Both graphs in Figure 4a,b have slopes greater than 1, indicating that there is a bias. This may mean that the complexometric values are overestimated or that the AAS values are underestimated. The latter is more likely because it is difficult to completely eliminate interference from phosphate ions. The bias is greater for sausages (Figure 4a) compared to MSM (Figure 4b) because sausages typically contain phosphate additives. 

The results of CDTA titration and ICP-OES measurement were also compared. Calcium was the second most abundant element in MSM (Table 3). The ratio of calcium to magnesium content is 8.5 which is characteristic of MSM samples [14]. Calcium content of 1560 ppm by ICP-OES (Table 3) is consistent with 1728 ppm measured by CDTA titration.

The complexometric titration technique was tested for robustness by varying the dosing rate and sample dilution. No bias in the results was recorded when the dosing rate ranged from 0.1 to 0.4 mL per minute and the sample solution volume ranged from 50 to 200 mL. The lower sample volume is beneficial because less indicator is used for the same indicator concentration. The effect of variations in sample mineralization temperature was also studied. An adequate temperature of the muffle furnace was found to be 600 °C. Increasing the mineralization temperature to 650 °C did not change the calcium content. On the other hand, lowering the temperature to 550 °C caused a decrease in the calcium content, as measured (Table 4).

### 2.2. Calcium Content in Commercial Bologna-Type Sausages

**Figure 5:**
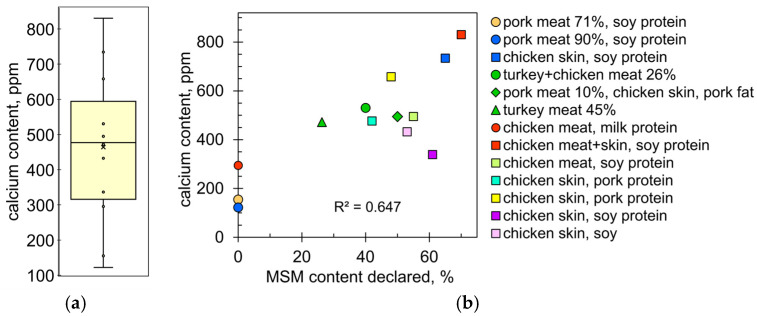
(**a**) Distribution of calcium content values in the sausage samples. The boxes indicate the upper and lower quartiles; the whiskers indicate the minimum and maximum values; and the × sign indicates the mean value. (**b**) Calcium content as a function of MSM content in the sausage samples. Meat producers are color coded. Ingredients other than MSM are listed in the legend.

Figure 5a summarizes the calcium content in the sausage samples. Two conclusions can be drawn: the values are scattered over a wide range, from 122 to 830 ppm, and the values are significantly higher than the calcium content in chicken meat, which is between 50 and 80 ppm [44,45,46]. The first obvious explanation is that sausages contain MSM, which is rich in calcium. However, there was no close correlation between the amount of calcium and the MSM content declared by the meat producers (Figure 5b). The value of the coefficient of determination is quite low (R^2^ = 0.647), which indicates practically no correlation. This can be explained by the fact that sausage manufacturers use different types of MSM. For example, the calcium content of the MSM samples varied widely, from 62 to 2833 ppm (Figure 4b). The first value corresponds exactly to pure meat while the second corresponds to high-pressure MSM. According to EU regulations, a meat product is considered a high-pressure MSM if the calcium content is 1000 ppm or more [5]. Of course, meat producers select the type and content of MSM in such a way that the calcium content in the final product does not exceed the threshold value of 1000 ppm (Figure 5a). 

**Figure 6:**
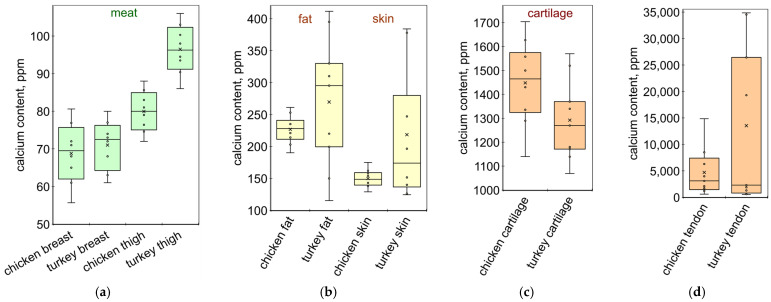
Calcium content in chicken and turkey parts: (**a**) meat; (**b**) fat and skin; (**c**) articular cartilage; and (**d**) tendon. The boxes indicate the upper and lower quartiles; the whiskers indicate the minimum and maximum values; and the × sign indicates the mean value.

MSM is not the only source of higher calcium levels. For this reason, the sausages without MSM also contained high amounts of calcium, i.e., 122, 155, and 295 ppm (Figure 5b). In these cases, the additional source of calcium was milk protein or soy protein. Sausage labels also list other calcium-rich ingredients, such as pork protein and chicken skin (Figure 5b). Chicken fat, skin, and paw cartilage are typical ingredients of Bologna-type sausages [52,53,54]. In fact, industrial sausage products often contain some amounts of finely ground poultry parts such as feet, tails, necks, combs, wattles, blood vessels, tendons, nerves, pygostyles, etc. [55]. For a more detailed picture, the calcium content of chicken and turkey parts from different producers was determined (Figure 6). 

Meat samples have typical calcium content: breast meat contains 56 to 81 ppm and thigh meat has 72 to 106 ppm (Figure 6a). Fat and skin samples have significantly more calcium (Figure 6b). The values of calcium content in turkey fat and skin vary widely, from 115 to 412 ppm, possibly due to the use of very different feeds by turkey producers. Articular cartilage samples contain as much as 1069–1704 ppm of calcium (Figure 6c). The highest calcium content was recorded in the samples of tendons, with the values varying in the very wide range of 532 to 34,539 ppm (Figure 6d). In fact, calcification along the tendon is uneven: the parts of the tendon close to the bone are more calcified compared to the parts close to the muscle. Thus, even a small amount of highly calcified tendon tissue can cause a significant increase in the calcium content of sausage material.

### 2.3. Compliance with the Principles of Green Analytical Chemistry

The results confirm that CDTA and TEA are very suitable reagents for the complexometric determination of calcium in the presence of phosphate ions. The dye Arsenazo III proved to be a good endpoint indicator for automatic titration with the Optrode immersion probe. In the next step, the CDTA titration procedure’s compliance with the concept of Green Analytical Chemistry was assessed. Table 5 summarizes the criteria to be met in order to obtain green status as well as the corresponding scores calculated using dedicated software (https://git.pg.edu.pl/p174235/AGREE, accessed on 10 September 2023) [56]. The weight coefficients were selected considering that the CDTA titration procedure is to be used in the meat industry. The overall score of greenness was found to be 0.6.

The procedure scored high on criteria 4, 5, 6, 11, and 12, with corresponding dark green and light green segments (Table 5). The scores for criteria 1, 2, 7, 8, and 9 were lower and the corresponding segments are yellow and orange. There were null scores for criteria 3 and 10 with corresponding red segments. Criteria 3, 4, 5, and 11 have increased weight coefficients due to their importance in the meat industry. A minimum procedural steps (criterion 4) reduces labor costs while a lack of toxic reagents (criterion 11) ensures a clean working environment. The main disadvantage of the proposed procedure is the necessary pre-treatment step, which resulted in low scores on criteria 1 and 3.

**Figure 7:**
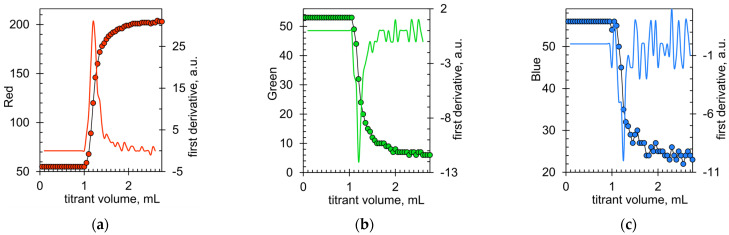
Changes in (**a**) red, (**b**) green, and (**c**) blue color components in the images of the reaction beaker were recorded during the complexometric determination of calcium in 1 g of a sample of brand I turkey sausage. The indicator was Arsenazo III at a concentration of 2 μM.

The AGREE index [56] does not take into account most of the economic aspects of the analytical procedure (only energy consumption in criterion 9). However, economic considerations are important for small enterprises in the meat industry. The titration technique is a very suitable methodology in this respect due to the low cost of reagents and labor. A further improvement in this direction could be made by using an optical sensor outside the titration beaker instead of an immersion probe. For example, the Cromlaview CR100 industrial color sensor was successfully used in turbidimetric titration [57,58]. The external optical sensor does not come into contact with the test solutions, so it does not require cleaning. In this work, a webcam was tested as an external instrument to detect the color change in Arsenazo III as an alternative to the immersion optical probe. Figure 7 shows that all the color components (i.e., red, green, and blue) in the images of the reaction beaker captured by the webcam during the titration changed rapidly at the endpoint. The titration plots had better shapes than those recorded when analyzing the same sausage with the immersion probe (Figure 3b). This is due to the RGB signals being less sensitive to the turbidity of the solution. The best signal is the red component, which ensures minimal fluctuations of the first derivative (Figure 7a). Thus, complexometric titration with Arsenazo III and automatic endpoint detection via webcam or smartphone could be a viable method for routine calcium determination in the meat industry. On the other hand, signals from cameras are sensitive to changes in lighting. Therefore, it is necessary to reliably test typical RGB cameras in various lighting conditions. This is a future area of research. 

## 3. Materials and Methods

### 3.1. Reagents and Meat Samples 

The reagents were spectrally pure hydrochloric acid (Merck, Rahway, NJ, USA), reagent-grade 1,2-diaminocyclohexane-N,N,N’,N’-tetraacetic acid, Arsenazo III dye, lanthanum chloride, certified calcium chloride solution (Aldrich, St. Louis, MO, USA), and reagent-grade triethanolamine and sodium hydroxide (POCh, Gliwice, Poland). Poultry meat and Bologna-type sausages were purchased from local grocery stores. Mechanically separated meat samples were obtained directly from meat producers. 

### 3.2. Procedures and Methods

UV–vis spectra were recorded using a Spectroquant Pharo 300 spectrophotometer (Merck) and quartz cuvettes with an optical path of 10 mm.

The meat products were tested within 1 day of collection. The sausages were peeled and homogenized in a standardized manner [59]. The samples for analysis were accurately weighed (±0.0001 g) and mineralized in a muffle furnace at 600 °C for 4 h. The resulting ash was dissolved in 5 mL of 30% hydrochloric acid and left for 24 h to hydrolyze polyphosphates [60].

For the titration procedure, the solutions were diluted with ultrapure water to a volume of 100 mL and alkalized to pH 12.5. Titration with 0.01 M CDTA was performed using a 751 GPD Titrino titrator (Metrohm, Herisau, Switzerland) and a Titronic 500 digital burette (SI Analytics, Weilheim, Germany). Color changes were registered using an Optrode^®^ photometric immersion probe (Metrohm) and a Logitech C270 webcam. The variance was determined based on 10 repeated determinations.

Reference determinations were made using an iCE 3000 SERIES atomic absorption spectrometer (Thermo Scientific, Waltham, MA, USA) with an acetylene–air flame and a calcium hole cathode lamp (422.7 nm). The sample solutions were diluted (1:100) and lanthanum chloride (1 g) was added to bind phosphate ions, which reduces the ionization of calcium in AAS plasma [61]. The calibration line was built using a certified calcium chloride solution (1000 ppm). Reference determinations by ICP-OES were performed on the spectral line of calcium at 317.933 nm using a spectrometer Spectro Arcos (Spectro Analytical Instruments GmbH, Kleve, Germany) with a dilution of 1:100 and flow rate of 2 mL/min.

## 4. Conclusions

The complexometric method for determining calcium with EDTA as a titrant is sensitive to phosphate ions in the sample. Therefore, for the analysis of meat products, instead of EDTA, 1,2-diaminocyclohexane-N,N,N’,N’-tetraacetic acid was used. Arsenazo III was selected as the most appropriate indicator. The results of the CDTA titration procedure were verified by comparison with AAS and ICP OES measurements.

The calcium content of industrial meat ingredients varies widely: from 62 to 2833 ppm in MSM, from 115 to 412 ppm in poultry fat and skin, from 1069 to 1704 ppm in articular cartilage, and from 532 to 34,539 ppm in tendon. That is why the calcium content in commercial Bologna-type sausages ranges from 122 to 830 ppm, which is visibly more than in pure meat.

This CDTA titration procedure was assessed against the Green Analytical Chemistry criteria and the overall score of greenness was found to be 0.6. The CDTA titration procedure is simple, reliable, and well suited to small meat processing plants.

## Figures and Tables

**Table 1:** Methods for measuring calcium in meat products.

Technique	Sample Preparation	Advantage	Disadvantage	Performance * [Ref.]
EDTA titration	acid digestion	simple instrumentation	interference from phosphates	RSD = 3.4% [21]
AAS	acid digestion	no interference from phosphates	cost of gases	RSD = 9% [13]
ICP-OES	acid digestion	multi-element determination	complicated instrumentation	RSD = 3.5–4.4% [24]
ICP-MS	acid digestion	multi-element determination	complicated instrumentation	RSD = 3.7% [25]
ion chromatography	acid digestion	simultaneous determination of Mg	no information	LOQ = 1.4 ppm [14]
LIBS	drying, pelletizing	multi-element determination	drying for 72 h	RSD = 8.5–8.9% [24]
LIBS	no	operates at a distance of 5 cm	uneven surface affects	RMSECV = 170 ppm [23]
Raman spectroscopy	no	operates at a distance of 25 cm	indirect determination	RMSECV = 3330 ppm [15]
TXRF	homogenization	multi-element determination	sample inhomogeneity affects	LOD = 0.93 ppm [16]

* RSD—relative standard deviation; LOQ—limit of quantitation; RMSECV—root mean square error of cross validation; LOD—limit of detection.

**Table 2:** Relative standard deviation of calcium determination in meat products.

Sample	Ca Content, ppm	RSD, %
chicken drumstick meat, brand W	125	3.1
chicken drumstick skin, brand W	149	12.6
chicken sausage, brand T	662	2.6
chicken sausage, brand KH	465	9.7
chicken sausage, brand D	791	7.4
chicken sausage, brand B	685	3.8
turkey MSM, brand G	60	5.7
chicken MSM, brand K	450	8.9
chicken MSM, brand D	2008	4.5

**Table 3:** The content of macroelements in brand D2 MSM determined by the ICP-OES method.

Element	Wavelength, nm	Content, ppm
K	769.896	2300
Ca	317.933	1560
Na	589.592	825
S	182.034	194.5
Mg	280.27	183.5

**Table 4:** Results of calcium determination in chicken sausage (brand B) mineralized at different temperatures.

Temperature, °C	Ca Content, ppm
650	690
600	685
550	520

**Table 5:** The principles of Green Analytical Chemistry and matching scores.

No.	Principle	Weight	Score	Overall Diagram
1	Direct analytical techniques should be applied to avoid sample treatment	2	0.3	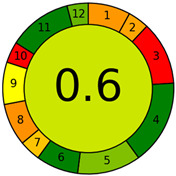
2	Minimal sample size and minimal number of samples are goals	1	0.32	
3	In situ measurements should be performed	3	0	
4	Integration of analytical operations saves energy and reduces the use of reagents	4	1	
5	Automated and miniaturized methods should be selected	3	0.75	
6	Derivatization should be avoided	2	1	
7	Generation of a large volume of analytical waste should be avoided	1	0.39	
8	Multianalyte or multiparameter methods are preferred	2	0.29	
9	The use of energy should be minimized	2	0.5	
10	Reagents obtained from renewable source should be preferred	1	0	
11	Toxic reagents should be eliminated or replaced	3	1	
12	The safety of the operator should be increased	2	0.8	

The colors correspond to the “greenness” of the individual criteria for the analytical method: from red (worst result) to dark green (best result).

## Data Availability

All data are contained within the article.

## Sample Availability

Meat product samples are no longer available due to their limited storage time.

## References

[1] Paglarini C.S., Vidal V.A.S., Neri-Numa I.A., Pastore G.M., Pollonio M.A.R. (2023). Effect of commercial plant extracts on the oxidative stability of mechanically deboned poultry meat during chilled storage. Food Res. Int..

[2] Kim J., Shand P.J. (2022). Combined effect of beet powder and lentil flour as a partial nitrite substitute on physicochemical, texture and sensory characteristics, color, and oxidative stability of pork bologna. J. Food Sci..

[3] Thames H.T., Fancher C.A., Colvin M.G., McAnally M., Tucker E., Zhang L., Kiess A.S., Dinh T.T.N., Sukumaran A.T. (2022). The Prevalence of Salmonella and Campylobacter on Broiler Meat at Different Stages of Commercial Poultry Processing. Animals.

[4] Cegiełka A., Chmiel M., Hać-Szymańczuk E., Pietrzak D. (2022). Evaluation of the Effect of Sage (*Salvia officinalis* L.) Preparations on Selected Quality Characteristics of Vacuum-Packed Chicken Meatballs Containing Mechanically Separated Meat. Appl. Sci..

[5] EFSA (European Food Safety Authority) (2013). Scientific opinion on the public health risks related to mechanically separated meat (MSM) derived from poultry and swine. EFSA J..

[6] Iammarino M., Miedico O., Petrella A., Mangiacotti M., Chiaravalle A.E. (2020). Innovative approaches for identifying a mechanically separated meat: Evaluation of radiostrontium levels and development of a new tool of investigation. J. Food Sci. Tech..

[7] Nagdalian A.A., Rzhepakovsky I.V., Siddiqui S.A., Piskov S.I., Oboturova N.P., Timchenko L.D., Lodygin A.D., Blinov A.V., Ibrahim S.A. (2021). Analysis of the content of mechanically separated poultry meat in sausage using computing microtomography. J. Food Compos. Anal..

[8] Tomaiuolo M., Chiaravalle A.E., Mangiacotti M., Petrella A., di Taranto A., Iammarino M. (2019). Innovative techniques for identifying a mechanically separated meat: Sample irradiation coupled to electronic spin resonance. Eur. Food Res. Technol..

[9] Branscheid W., Judas M., Höreth R. (2009). The morphological detection of bone and cartilage particles in mechanically separated meat. Meat Sci..

[10] Pospiech M., Zikmund T., Javůrková Z., Kaiser J., Tremlová B. (2019). An Innovative Detection of Mechanically Separated Meat in Meat Products. Food Anal. Method.

[11] Kiełczyński P., Szymański P., Szalewski M., Wieja K., Balcerzak A., Ptasznik S. (2022). Application of Density Measurements for Discrimination and Evaluation of Chemical Composition of Different Types of Mechanically Separated Meat (MSM). Molecules.

[12] Wieja K., Kiełczyński P., Szymański P., Szalewski M., Balcerzak A., Ptasznik S. (2021). Identification and investigation of mechanically separated meat (MSM) with an innovative ultrasonic method. Food Chem..

[13] Mohamed M.A., Kassem G.M., Zahran D.A., Emara M.T., Mansour N.K. (2023). Impact of mechanically recovered poultry meat (MRPM) on proximate analysis and mineral profile of traditional Egyptian luncheon. J. Radiat. Res. Appl. Sci..

[14] Iammarino M., Miedico O., Sangiorgi E., D’Amore T., Berardi G., Accettulli R., Dalipi R., Marchesani G., Chiaravalle A.E. (2021). Identification of mechanically separated meat in meat products: A simplified analytical approach by ion chromatography with conductivity detection. Int. J. Food Sci. Technol..

[15] Wubshet S.G., Wold J.P., Böcker U., Sanden K.W., Afseth N.K. (2019). Raman spectroscopy for quantification of residual calcium and total ash in mechanically deboned chicken meat. Food Control.

[16] Dalipi R., Berneri R., Curatolo M., Borgese L., Depero L.E., Sangiorgi E. (2018). Total reflection X-ray fluorescence used to distinguish mechanically separated from non-mechanically separated meat. Spectrochim. Acta Part B At. Spectrosc..

[17] Wilhelm C., Hofsommer M., Wittke S. (2022). Detection of Mechanically Separated Meat from Chicken in Sausages and Cold Meat by Targeted LC–MS/MS Analysis. Food Anal. Method.

[18] Monago-Maraña O., Wold J.P., Rødbotten R., Dankel K.R., Afseth N.K. (2021). Raman, near-infrared and fluorescence spectroscopy for determination of collagen content in ground meat and poultry by-products. LWT.

[19] Meat and Poultry Labeling Terms (2015). Food Safety and Inspection Service.

[20] Corrao P.A., Malanoski A.J., Curry K.A., Glover A. (1983). Titrimetric Determination of Calcium in Mechanically Separated Poultry and Beef: Collaborative Study. J. AOAC Int..

[21] Tasić A., Kureljušić J., Nešić K., Rokvić N., Vićentijević M., Radović M., Pisinov B. (2017). Determination of calcium content in mechanically separated meat. IOP Conf. Ser. Earth Environ. Sci..

[22] Poitevin E. (2012). Determination of Calcium, Copper, Iron, Magnesium, Manganese, Potassium, Phosphorus, Sodium, and Zinc in Fortified Food Products by Microwave Digestion and Inductively Coupled Plasma-Optical Emission Spectrometry: Single-Laboratory Validation and Ring Trial. J. AOAC Int..

[23] Andersen M.-B.S., Frydenvang J., Henckel P., Rinnan Å. (2016). The potential of laser-induced breakdown spectroscopy for industrial at-line monitoring of calcium content in comminuted poultry meat. Food Control.

[24] Leme F.O., Silvestre D.M., Nascimento A.N., Nomura C.S. (2018). Feasibility of using laser induced breakdown spectroscopy for quantitative measurement of calcium, magnesium, potassium and sodium in meat. J. Anal. Atom. Spectrom..

[25] Miedico O., Nardelli V., D’Amore T., Casale M., Oliveri P., Malegori C., Paglia G., Iammarino M. (2022). Identification of mechanically separated meat using multivariate analysis of 43 trace elements detected by inductively coupled mass spectrometry: A validated approach. Food Chem..

[26] de Silva S.M., Deraniyagala S., Walpita J.K., Jayaweera I., Diyabalanage S., Cooray A.T. (2020). Masking Ability of Various Metal Complexing Ligands at 1.0 mM Concentrations on the Potentiometric Determination of Fluoride in Aqueous Samples. J. Anal. Methods Chem..

[27] Huang W., Shi Y., Yan H., Wang H., Wu D., Grierson D., Chen K. (2023). The calcium-mediated homogalacturonan pectin complexation in cell walls contributes the firmness increase in loquat fruit during postharvest storage. J. Adv. Res..

[28] Peng X., Liu J., Tang N., Deng J., Liu C., Kan H., Zhao P., Zhang X., Shi Z., Liu Y. (2023). Sequential extraction, structural characterization, and antioxidant activity of polysaccharides from *Dendrocalamus brandisii* bamboo shoot shell. Food Chem. X.

[29] Sun X., Wang P., Shen X., Chen F., Zhang L. (2023). Changes of Ca forms and chelate-soluble pectin in cherry tomatoes treated with ultrasound and calcium lactate. LWT.

[30] Isager Ahl L., Pedersen H.L., Jørgensen B., Willats W.G.T., Grace O.M., Barnes C.J., Rønsted N. (2023). Exploring the polysaccharide composition of plant cell walls in succulent aloes. Plants People Planet.

[31] Bellinger B.J., McKenney E.L., Gretz M.R. (2023). Identifying plant cell wall remnants in detritus of a subtropical wetland with fluorescence labeling. Geoderma.

[32] Pan X., Zhao W., Wang Y., Xu Y., Zhang W., Lao F., Liao X., Wu J. (2022). Physicochemical and structural properties of three pectin fractions from muskmelon (*Cucumis melo*) and their correlation with juice cloud stability. Food Hydrocoll..

[33] Yu C., Ahmadi S., Shen S., Wu D., Xiao H., Ding T., Liu D., Ye X., Chen S. (2022). Structure and fermentation characteristics of five polysaccharides sequentially extracted from sugar beet pulp by different methods. Food Hydrocoll..

[34] Yu C., Hu X., Ahmadi S., Wu D., Xiao H., Zhang H., Ding T., Liu D., Ye X., Chen S. (2022). Structure and In Vitro Fermentation Characteristics of Polysaccharides Sequentially Extracted from Goji Berry (*Lycium barbarum*) Leaves. J. Agric. Food Chem..

[35] Wehr J.B., Dalzell S.A., Menzies N.W. (2023). Predicting and modelling availability of fluoride in soil from sorption properties. Soil Use Manag..

[36] Jordan D.E., Monn D.E. (1967). Rapid determination of magnesium in the presence of calcium and phosphate by titration with cdta. Anal. Chim. Acta.

[37] Tuckerman M.M., Sanchez de Ramos M.E. (1977). Direct Complexometric Titration of Calcium Phosphates. J. Pharm. Sci..

[38] Kapeluszna E., Chrabąszcz K. (2023). Mutual compatibility of superplasticizers (PC, SNF), grinding aids (TEA, glycol) and C_3_A in Portland cement systems—Hydration, rheology, physical properties and air void characteristics. Constr. Build. Mater..

[39] Kirchberger I., Goetz-Neunhoeffer F., Neubauer J. (2023). Enhancing the aluminate reaction during OPC hydration by combining increased sulfate content, triethanolamine and tartaric acid. Cem. Concr. Res..

[40] Jiang J., Liu B., Shi J., Gencel O., An X., Gao J. (2022). Synergistic effect of glycine and triethanolamine on mechanical properties and permeability of cement mortar. J. Build. Eng..

[41] Zhang W., Sun Y., Yang Y., Chen Y. (2023). Impaired intracellular calcium homeostasis enhances protein O-GlcNAcylation and promotes vascular calcification and stiffness in diabetes. Redox Biol..

[42] Karavasiloglou N., Hughes D.J., Murphy N., Schomburg L., Sun Q., Seher V., Rohrmann S., Weiderpass E., Tjønneland A., Olsen A. (2023). Prediagnostic serum calcium concentrations and risk of colorectal cancer development in 2 large European prospective cohorts. Am. J. Clin. Nutr..

[43] Kalavathy N., Anantharaj N., Sharma A., Chauhan T. (2022). Effect of serum vitamin D, calcium, and phosphorus on mandibular residual ridge resorption in completely edentulous participants: A clinical study. J. Prosthet. Dent..

[44] Orkusz A. (2021). Edible Insects versusMeat—Nutritional Comparison: Knowledge of Their Composition Is the Key to Good Health. Nutrients.

[45] Dehelean A., Cristea G., Puscas R., Hategan A.R., Magdas D.A. (2022). Assigning the Geographical Origin of Meat and Animal Rearing System Using Isotopic and Elemental Fingerprints. Appl. Sci..

[46] Islam M.A., Jeong J.Y., Kim E.J., Khan N., Jamila N., Kim K.S. (2023). Multielemental Characterization of Chicken Breasts from Conventional and Sustainable Farms by Inductively Coupled Plasma—Optical Emission Spectrometry (ICP-OES) and Inductively Coupled Plasma—Mass Spectrometry (ICP-MS). Anal. Lett..

[47] Araujo-Chapa A.P., Urías-Orona V., Niño-Medina G., Muy-Rangel D., de la Garza A.L., Castro H. (2023). Dietary Fiber from Soybean (Glycine max) Husk as Fat and Phosphate Replacer in Frankfurter Sausage: Effect on the Nutritional, Physicochemical and Nutraceutical Quality. Molecules.

[48] Powell M.J., Sebranek J.G.G., Prusa K.J., Tartéa R. (2019). Evaluation of citrus fiber as a natural replacer of sodium phosphate in alternatively-cured all-pork Bologna sausage. Meat Sci..

[49] Magalhães I.M.C., Paglarini C.S., Vidal V.A.S., Pollonio M.A.R. (2020). Bamboo fiber improves the functional properties of reduced salt and phosphate-free Bologna sausage. J. Food Process Preserv..

[50] Jonauske V., Stanionyte S., Chen S.-W., Zarkov A., Juskenas R., Selskis A., Matijosius T., Yang T.C.K., Ishikawa K., Ramanauskas R. (2019). Characterization of Sol-Gel Derived Calcium Hydroxyapatite Coatings Fabricated on Patterned Rough Stainless Steel Surface. Coatings.

[51] Gaafar M.S., Yakout S.M., Barakat Y.F., Sharmoukh W. (2022). Electrophoretic deposition of hydroxyapatite/chitosan nanocomposites: The effect of dispersing agents on the coating properties. RSC Adv..

[52] Peña-Saldarriaga L.M., Pérez-Alvarez J.A., Fernández-López J. (2020). Quality Properties of Chicken Emulsion-Type Sausages Formulated with Chicken Fatty Byproducts. Foods.

[53] Peña-Saldarriaga L.M., Fernández-López J., Pérez-Alvarez J.A. (2020). Quality of Chicken Fat by-Products: Lipid Profile and Colour Properties. Foods.

[54] Lima J.L., Assis B.B.T., Olegario L.S., Galvão M.S., Soares Á.J., Arcanjo N.M.O., González-Mohino A., Bezerra T.K.A., Madruga M.S. (2021). Effect of adding byproducts of chicken slaughter on the quality of sausage over storage. Poult. Sci..

[55] Baéza E. (2020). Characteristics of processed poultry products. Worlds Poult. Sci. J..

[56] Pena-Pereira F., Wojnowski W., Tobiszewski M. (2020). AGREE—Analytical GREEnness Metric Approach and Software. Anal. Chem..

[57] Ziółkowska D., Syrotynska I., Shyichuk A., Lamkiewicz J. (2021). Determination of SLES in Personal Care Products by Colloid Titration with Light Reflection Measurements. Molecules.

[58] Ziółkowska D., Lamkiewicz J., Shyichuk A. (2022). Structure and Flocculation of Ion Associates of Carrageenan and Poly(diallyldimethylammonium chloride) Depending on the Component Ratio. Molecules.

[59] (2013). Foodstuffs—Determination of Elements and Their Chemical Species—General Considerations and Specific Requirements.

[60] (2003). Foodstuffs—Determination of Lead, Cadmium, Zinc, Copper, Iron and Chromium by Atomic Absorption Spectrometry (AAS) after Dry Ashing.

[61] (2002). Water Quality—Determination of Calcium and Magnesium—Atomic Absorption Spectrometric Method.

